# Corrosion Behavior of Ti_3_SiC_2_ in Flowing Liquid Lead–Bismuth Eutectic at 500 °C

**DOI:** 10.3390/ma15217406

**Published:** 2022-10-22

**Authors:** Liangliang Lyu, Xi Qiu, Huifang Yue, Mingyang Zhou, Huiping Zhu

**Affiliations:** 1 Science and Technology on Reactor System Design Technology Laboratory, Nuclear Power Institute of China, No. 328, Section 1, Changshun Avenue, Huayang, Shuangliu District, Chengdu 610213, China; 2 School of Nuclear Science and Engineering, North China Electric Power University, Beijing 102206, China

**Keywords:** MAX phase, LBE corrosion, corrosion structure, microhardness, wettability

## Abstract

MAX phases are promising candidate structural materials for lead-cooled fast reactors (LFRs) and accelerator-driven sub-critical systems (ADSs) due to their excellent corrosion resistance in liquid LBE. In this work, one of the typical MAX phases, Ti_3_SiC_2_, was exposed to the flowing LBE with a saturated oxygen concentration at 500 °C for up to 3000 h. The corrosion behaviors, including the evolution of the corrosion layer, mechanical properties and wettability, were evaluated via X-ray diffraction, a scanning electron microscope equipped with an energy-dispersive X-ray, a microhardness test and contact angle measurement. The results reveal that a corrosion structure with a duplex layer was formed on the sample surfaces. The outer layer was a diffusion layer, which always remained thin (<3 μm) during the whole test due to the erosion effect caused by the flowing LBE. The inner layer was the stable protective oxide layer, and its thickness increased with exposure time. The growth of the corrosion structure improved the microhardness and reduced the wettability with regard to LBE, which was beneficial to inhibiting further surface corrosion of Ti_3_SiC_2_.

## 1. Introduction

Lead-based alloys, which have a low melting point, high thermal conductivity, excellent neutron science performance and good chemical stability, are used as coolant materials for lead-cooled fast reactors (LFRs) and accelerator-driven sub-critical systems (ADSs). However, lead-based alloys have a strong corrosive effect on structural materials in the actual operating environment, which might threaten the safe operation of the reactor [1]. At present, developing novel structural materials using lead-based alloys with excellent corrosion resistance is one of the most important ways to solve this problem.

The MAX phases are ternary carbides and nitrides described by the general formula M_n+1_AX_n_ (n = 1, 2 or 3), where M is an early transition metal, A denotes an element from groups 13–16 in the periodic table of elements, and X is either C or N [2,3,4]. As typical metal–ceramic materials, the MAX phases have excellent physical and mechanical properties, such as an excellent oxidation resistance, high thermal conductivity, good machinability and irradiation resistance. Most of all these materials also exhibit promising resistance to liquid metal corrosion (LMC) and/or erosion when in contact with liquid Pb/LBE, which are considered as some of the most important candidate structural materials or coating materials for LFRs and ADSs at present [5]. 

Nowadays, most studies on MAX phase materials are focused on the irradiation effect [6,7,8], while the research on LBE corrosion performance is insufficient. Although researchers have investigated the corrosion behavior of many kinds of MAX phases in LBE, including Ti_3_SiC_2_, Ti_2_AlC, Ti_3_AlC_2_, (Ti, Nb)_2_AlC, Nb_2_AlC, Nb_4_AlC_3_, (Nb_0.85_, Zr_0.15_)_4_AlC_3_, Zr_2_AlC, Zr_3_AlC_2_, Ti_2_SnC, (Zr_0.8_, Ti_0.2_)_2_AlC and (Zr_0.8_, Ti_0.2_)_3_AlC_2_ [9,10,11], the research is not systematic enough, and the corrosion mechanisms differ with different kinds of MAX phase materials. Among these materials, Ti_3_SiC_2_ is one of the most studied and typical MAX phase materials with known properties, which have also attracted much attention due to the LBE corrosion resistance they confer. Rivai and Takahashi [12] found that Ti_3_SiC_2_ presented good compatibility with low-oxygen-containing (*C_O_* ≈ 5 × 10^−6^ wt%) LBE at 700 °C for up to 1000 h. Barnes et al. [13] have investigated the corrosion behavior of Ti_3_SiC_2_ in liquid Pb circulating by natural convection at 650 °C and 800 °C for 1000 h, and no obvious LMC damage was observed. Utili et al. [14] exposed Ti_3_SiC_2_ to flowing liquid Pb (*v* ≈ 1 m/s, *C_O_* ≈ 10^−6^ wt%) at 500 °C for 1000 h, and they also did not observe any LMC attack on the experimental materials. Heinzel et al. [15] studied the corrosion performance of Ti_3_SiC_2_ in static liquid LBE containing two different oxygen concentrations (*C_O_* ≈ 10^−6^ wt% and *C_O_* ≈ 10^−8^ wt%) at 550 °C to 700 °C for long-term exposure (up to 10,000 h). The results reveal that the contents of the corrosion products and corrosion structure deeply depended on the exposure temperature and oxygen concentration. For example, at the condition of 550 °C and *C_O_* ≈ 10^−6^ wt%, thin TiO_2_ oxide scales formed at the Ti_3_SiC_2_ surface; however, when the temperature increased to 650 °C/700 °C with the same oxygen concentration, complex oxide scales (single-layer TiO_2_ outer scale, duplex SiO_2_/TiO_2_ inner scale) could be observed. In our previous study, Ti_3_SiC_2_ samples were exposed to static LBE with a saturated oxygen concentration (~6.3 × 10^−4^ wt%) at 500 °C, and a duplex corrosion structure consisting of a diffusion layer and oxide layer on the Ti_3_SiC_2_ surface was observed, and the formation mechanism of the corrosion structure was also proposed [16]. From the literature above, all studies on the exposure of Ti_3_SiC_2_ to Pb/LBE are focused on corrosion behavior, while other important properties, such as mechanical performance and wettability, have not been reported. 

Accordingly, Ti_3_SiC_2_ was exposed to flowing LBE at 500 °C for up to 3000 h to systematically investigate its corrosion behavior. The microstructure observation, microhardness analysis and wettability analysis were conducted to evaluate the effects of LMC via flowing LBE on the Ti_3_SiC_2_ surface. In addition, the main results in this work were compared to those obtained at static conditions in our previous study to determine the effects of the flowing LBE on the corrosion structure. Finally, the effects of the corrosion structure, especially the oxide layer, on the evolution of microhardness and wettability of the Ti_3_SiC_2_ surface were discussed in detail. 

## 2. Experimental Procedure

### 2.1. Materials and Preparation

The Ti_3_SiC_2_ samples in this work were fabricated using the spark plasma sintering (SPS) method. The Ti, Si and C powders were added into a milling container at the stoichiometric proportion of 3:1:2 and then milled and sintered at 1400 °C for 10 min under a constant pressure of 30 MPa in an Ar atmosphere. Additionally, all the samples were machined to 15 mm × 7.5 mm × 1 mm in size. Finally, the surfaces of the samples were mechanically ground using 1200#, 1500# and 2000# sandpapers in turn, polished to a mirror-like finish and ultrasonically cleaned in acetone and ethanol. The density of the obtained Ti_3_SiC_2_ was 4.28 g/cm^3^, which seemed to be relatively dense (about 94% T.D.). Additionally, the micrograph of the prepared Ti_3_SiC_2_ sample can be found in our previous work [16]. 

### 2.2. Corrosion Tests

**Figure 1:**
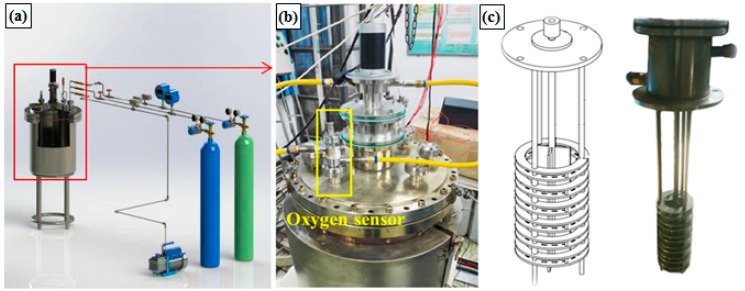
Schematic illustration of the pot-type multifunctional LBE corrosion platform: (**a**) corrosion tank, (**b**) the structure of oxygen sensor and (**c**) specimen holder.

Corrosion tests were conducted on a self-made pot-type multifunctional LBE corrosion platform, as shown in Figure 1. All samples were placed in specimen holders (in Figure 1c) and immersed in liquid LBE with a saturated oxygen concentration (~6.3 × 10^−4^ wt%) [17] at 500 °C. The specimen holders were rotated by an upper motor at the platform with a relative speed of 0.6 m/s between the samples and the liquid LBE. The samples were divided into six groups for 500 h, 1000 h, 1500 h, 2000 h, 2500 h and 3000 h, respectively. Three parallel specimens were used for each exposure parameter. Additionally, more information about the corrosion experiment can be found in our previous work [16].

### 2.3. Characterization and Analysis

After the corrosion tests, a scanning electron microscope (SEM, FEI HELIOS NANOLAB600, Hillsboro, OR, USA), energy-dispersive X-ray (EDX), X-ray diffraction (XRD, X’Pert^3^ereder) and Raman spectra (LabRam HR800) were used to analyze the microstructure, element distribution and phase evolution of the corroded samples, respectively. The XRD spectra were measured using a Cu-Kα radiation source with a scanning rate of 0.050/s. The Raman spectra were collected using a 532 nm wavelength laser at a typical laser power of 50 mW. Additionally, the microhardness of the samples before and after corrosion were also examined to characterize the hardness evolution of the material surface. The microhardness test was conducted on a Vickers hardness tester (Aiceyi, Dongguan, China), and the selected load was 1000 gf, with holding for 15 s. To improve the accuracy of the hardness measurement, the average values of the hardness in this work were calculated from at least seven measurements. In addition, the wettability of the corroded sample surface was also evaluated by measuring the contact angle at 500 °C.

It should be pointed out that in order to maintain the surface structure of the corroded samples, the samples used for the cross-section observation by SEM and EDX were directly inlayed with epoxy resin without cleaning. Additionally, the corroded samples for other experiments were initially cleaned in hot glycerol at 160 °C first, followed by a 20 s wash in a 50 wt% nitric acid solution to remove the remaining LBE from the surface.

## 3. Results and Discussion

### 3.1. Phase Analysis

**Figure 2:**
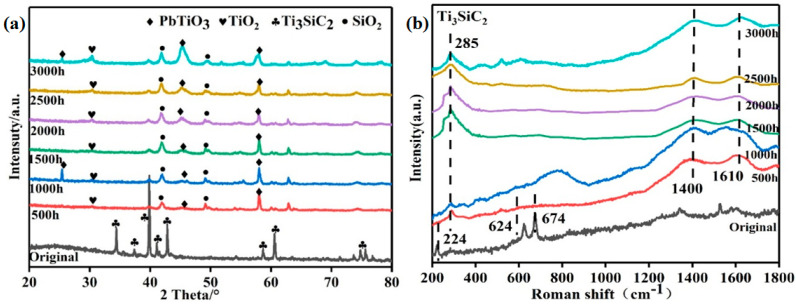
(**a**) XRD and (**b**) Raman results of Ti_3_SiC_2_ samples before and after LBE corrosion.

Figure 2 shows the XRD and Raman spectral results of the Ti_3_SiC_2_ samples before and after corrosion. In Figure 2a, it can be seen that additional oxides were observed for the corroded samples when compared with the pristine sample, indicating that the main corrosion products of Ti_3_SiC_2_ were PbTiO_3_, TiO_2_ and SiO_2_. Additionally, the intensity of the two main peaks for PbTiO_3_ gradually increased from 500 h to 3000 h, which might be ascribed to the further formation of PbTiO_3_ with the proceeding corrosion. In Figure 2b, a significant change in the Raman spectrum of the Ti_3_SiC_2_ samples exposed to LBE can be observed. Compared with the pristine sample, the corroded ones show a clear peak near the 285 cm^−1^ position, which corresponds to the corrosion product of PbTiO_3_ [18]. At the same time, two new peaks appeared at the position near 1400 and 1640 cm^−1^, which are the D and G peaks of carbon, respectively, indicating the formation of amorphous carbon and graphite [19,20]. These carbon-rich phases might be caused by the displacement of Ti and Si in Ti_3_SiC_2_ by carbon atoms due to the diffusion effect between LBE and Ti_3_SiC_2_, which can promote the migration of carbon atoms to the position of Ti and Si atoms [21,22]. With the prolonging of corrosion time, no other new peaks appeared in either the XRD patterns and Raman spectra, indicating that the phase composition of the corrosion structures on the surface of Ti_3_SiC_2_ remained stable in this work.

### 3.2. Microstructure of the Cross-Section

**Figure 3:**
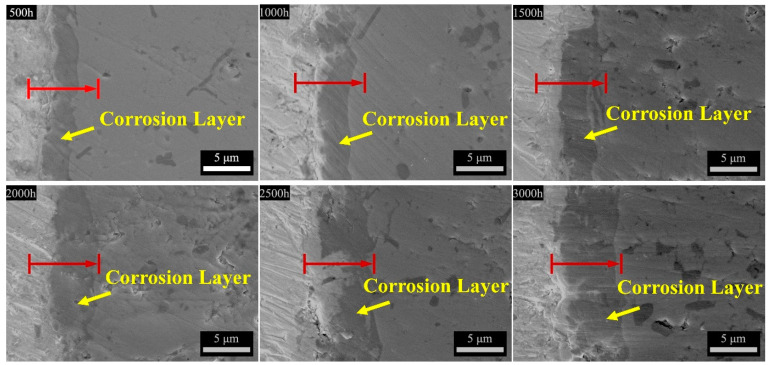
Cross-section morphology of Ti_3_SiC_2_ after LBE corrosion for 500 h to 3000 h (The red arrows in the figures are the scanning directions of EDX).

**Figure 4:**
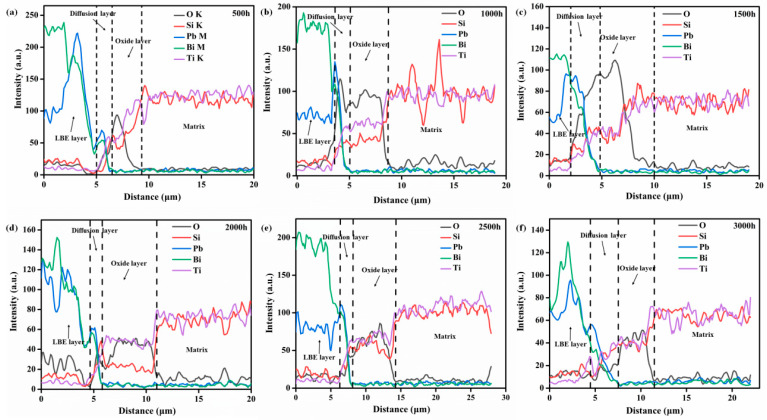
EDX line scan of cross-section of Ti_3_SiC_2_ after LBE corrosion for 500 h to 3000 h (**a**–**f**).

In order to understand the microstructural evolution and corrosion behavior, the cross-section morphology and composition distribution of the corroded Ti_3_SiC_2_ samples were characterized as shown in Figure 3 and Figure 4. Figure 3 exhibits the cross-section images of Ti_3_SiC_2_ samples exposed to LBE for different times. For all samples, a darker layer can be observed on the shallow Ti_3_SiC_2_ matrix. Additionally, the darker layer became thicker with prolonged exposure. Figure 4 presents the EDX line scan of exposed Ti_3_SiC_2_ samples corresponding to the red line in Figure 3. All the samples showed coincided signals of Pb, Bi, O, Ti and Si elements, and based on the distribution of these different elements, the corrosion cross-section can be divided into four layers. The outmost one is the uncleaned LBE region, namely the LBE layer. The innermost side is the unaffected Ti_3_SiC_2_ matrix, which will be defined as the matrix layer. The region located between the LBE layer and the matrix layer is the corrosion-affected zone, which can be divided into a diffusion layer and an oxide layer. The diffusion layer is close to the LBE layer, in which the content of oxygen elements is significantly higher than that of the Pb–bismuth layer, and the content of Pb and Bi elements gradually decreased with depth, while the Ti and Si elements gradually increased, indicating an obvious element diffusion phenomenon in this layer. The region near the matrix side is the oxide layer, in which the oxygen atoms diffused into the matrix begin to react with the matrix to form stable oxides. The diffusion layer and the oxide layer together constitute the corrosion layer of the exposed samples, leading to the formation of a typical duplex layer structure on the Ti_3_SiC_2_. In addition, combined with the XRD and Raman analysis results, it is clear that PbTiO_3_ is the dominant corrosion product in the diffusion layer, while the oxide layer mainly consists of TiO_2_ and SiO_2_.

Based on the element distribution of the corrosion layer for the same sample, the thickness of the diffusion layer, oxide layer and total corrosion layer can be obtained, respectively, by calculating the average thickness of ten typical locations, and the statistical results are shown in Table 1. It can be seen that the duplex corrosion structure formed after 500 h of exposure. Additionally, with the increase in exposure time, the thickness of both the diffusion layer and the oxide layer gradually increased, and 6.98 μm for the diffusion layer and 9.12 μm for the oxide layer were reached after 3000 h of exposure, respectively. However, the thickness of the diffusion layer never exceeded 3 μm and fluctuated with exposure time. 

Compared with the results of our previous work under static conditions [16], the evolution of the corrosion layers of Ti_3_SiC_2_ in this work exhibited different trends. In general, the thickness of the diffusion layer under static conditions was much greater than that of samples under the same conditions for different exposure times, while the oxide layer presented different trends. The oxide layer for samples exposed to static LBE obtained a stable thickness after 2000 h of exposure; however, a continuing increase trend was observed for the thickness of the oxide layer for samples corroded by flowing LBE. Additionally, a thicker oxide layer was obtained at the end of the flowing exposure experiment. The different corrosion behaviors of Ti_3_SiC_2_ in static and flowing LBE may have been caused by the different corrosion mechanisms. As for the static state, dissolution corrosion and oxidation were the dominant corrosion mechanisms, and the former one was responsible for the formation of a diffusion layer, while the latter one promoted the growth of an oxide layer. In addition to the above two mechanisms, the flow-assisted corrosion mechanism also played an important role in the formation of the corrosion layer for the flowing exposure. Unlike the growth pattern of the diffusion layer under static corrosion, the diffusion layer structure formed on the Ti_3_SiC_2_ surface was peeled off during the flushing process by the flow of LBE; thus, the thickness continuously maintained a thin state. The thinner diffusion layer was more conducive to the diffusion of oxygen elements to the oxide layer, and the flowing LBE could also supply enough dissolved oxygen to form an oxide layer, resulting in thicker oxide layers under flowing conditions. However, it should be pointed out that the flowing conditions created in this work may not fully reflect the actual operating conditions of the cooling system for LFRs or ADSs. Thus, the erosion and corrosion phenomena of the samples described in this work may differ from the results obtained under real conditions. 

### 3.3. Microhardness Analysis 

**Figure 5:**
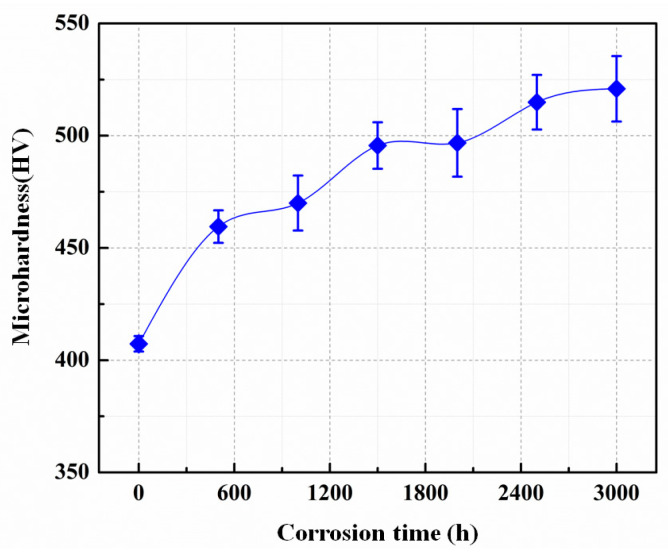
Surface microhardness of Ti_3_SiC_2_ sample corroded by LBE for 500–3000 h.

Figure 5 exhibits the microhardness evolution of the surface of Ti_3_SiC_2_ samples during exposure. It can be seen that the microhardness of the origin surface of the Ti_3_SiC_2_ sample was about 407 HV, and after corrosion, the value of the hardness increased from 460 HV to 620 HV as the exposure time increased from 500 h to 3000 h, indicating a significant surface hardening effect because of the generation of the corrosion layers. In addition, it is worth noting that after corrosion, the formation of the corrosion layer led to a reduction in the flatness of the material surface, resulting in a greater hardness error at different locations on the material surfaces.

It is clear that the hardness evolution trend was highly consistent with the growth trend of the thickness of the oxide layer and the total corrosion layer on the material surface, indicating that the formation of the corrosion layer likewise changes the mechanical properties of the Ti_3_SiC_2_ surface. With the growth of the corrosion layer, the microhardness values of the material surface gradually increased. For the diffusion layer structure on the outer surface, which mainly consisted of PbTiO_3_, the thickness did not change much with the prolongation of corrosion time due to the effect of surface scouring, while the thickness of the oxide layer, which consisted of TiO_2_ and SiO_2_, gradually increased. Thus, the main reason for the surface hardening of the material was the formation of surface corrosion layers, in which the thickening of the oxide layer played a major role.

### 3.4. Wettability Evaluation

**Figure 6:**
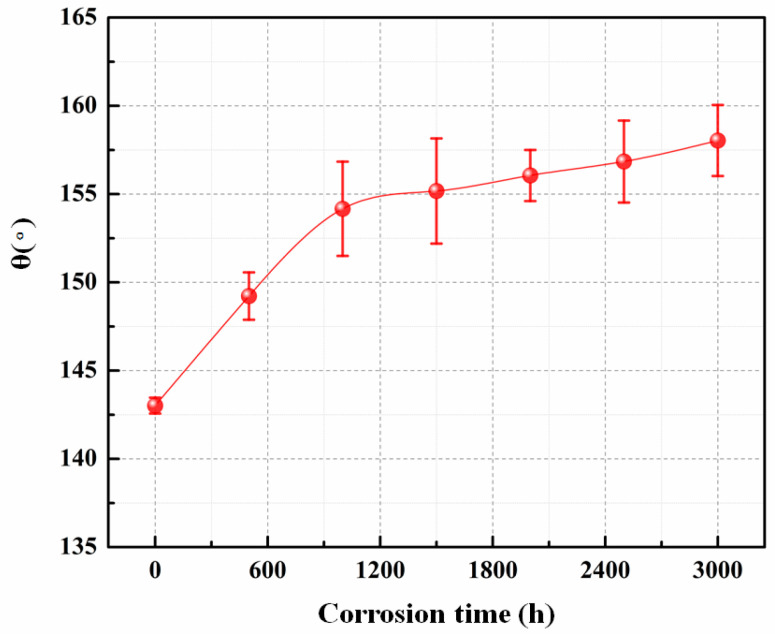
The wetting angle (θ) between LBE and Ti_3_SiC_2_ samples before and after corrosion at 500 °C.

Liquid metal embrittlement (LME), which is extremely dependent on the wettability between structural materials and liquid metal coolant, is a major concern for the safe operation of reactors in actual service environments. Additionally, the wettability can be evaluated via a solid–liquid infiltration experiment, which in turn reveals the LME tendency of the material. Currently, the wettability between solids and liquids is commonly characterized by the contact angle *θ* formed between the liquid material and the solid material. In this paper, the results for the contact angle *θ* between the Ti_3_SiC_2_ surface, before and after corrosion and LBE droplets were carried out at 500 °C, are shown in Figure 6. For the uncorroded sample, the contact angle of the Ti_3_SiC_2_ surface with an LBE temperature of 500 °C was 143.01°, indicating it is a non-infiltrated surface. After corrosion, the contact angle of the material surface gradually increased as the exposure time increased. The value of *θ* reached 154.16° after 1000 h of exposure, which is much greater than the boundary value of non-wettability and super non-wettability [23], indicating that the corrosion surface of Ti_3_SiC_2_ is super non-wettable to LBE. It is noteworthy that the trend of the change in the contact angle of the material surface to LBE after corrosion is also consistent with the growth trend of the oxide layer thickness and corrosion layer thickness in the previous section. The wettability of the Ti_3_SiC_2_ surface regarding the liquid metal was directly related to the nature of material surface, structure and roughness, and this phenomenon indicates that the corrosion layer on the Ti_3_SiC_2_ surface had higher non-wettability compared to the substrate itself. Thus, the formation of the corrosion layer isolates the Ti_3_SiC_2_ matrix from the LBE coolant to some extent, and its poorer wettability also slows down the LME tendency of the corroded samples and contributes to the reduction in the corrosion rate of the material surface.

## 4. Conclusions

In this work, the corrosion behavior of Ti_3_SiC_2_ in flowing LBE with saturated oxygen concentrations at 500 °C was systematically investigated via microstructure observation, microhardness analysis and wettability evaluation. Additionally, the following conclusions can be drawn from this work:

(1) The corrosion layer of Ti_3_SiC_2_ was an obvious duplex-layer structure containing an outer diffusion layer dominated by PbTiO_3_ and an inner oxide layer consisting of TiO_2_ and SiO_2_. 

(2) The flow-accelerated corrosion effect caused by the flowing LBE led to the dissolution and peeling of the diffusion layer, maintaining the thickness of the diffusion layer below 3 μm, and promoted the penetration of oxygen atoms into the matrix, resulting in a thicker oxide layer than that seen under the same conditions in the static LBE.

(3) The surface hardness of Ti_3_SiC_2_ gradually increased with the exposure time, and the hardness evolution trend was highly consistent with the growth trend of the thickness of the oxide layer and the total corrosion layer. 

(4) The wettability of LBE on Ti_3_SiC_2_ surface was reduced with the increase in exposure time, and it reached a super non-wettable state after only 1000 h of exposure, which can effectively decrease the LME tendency for Ti_3_SiC_2_.

## Figures and Tables

**Table 1:** The statistical thicknesses of the diffusion layer, oxide layer and corrosion layer of LBE-corroded Ti_3_SiC_2_ samples.

Corrosion Time (h)	Diffusion Layer Thickness (μm)	Oxide Layer Thickness (μm)	Corrosion Layer Thickness (μm)
500	1.32 (±0.51)	2.80 (±0.89)	4.12 (±1.40)
1000	1.50 (±0.68)	3.38 (±1.01)	4.88 (±1.69)
1500	2.73 (±0.67)	3.43 (±0.83)	6.16 (±1.50)
2000	2.52 (±0.72)	4.05 (±0.93)	6.57 (±1.65)
2500	1.81 (±0.73)	6.32 (±1.08)	8.13 (±1.81)
3000	2.14 (±0.87)	6.98 (±0.92)	9.12 (±1.79)
